# RoadMApp: a feasibility study for a smart travel application to improve maternal health delivery in a low resource setting in Zimbabwe

**DOI:** 10.1186/s12884-020-03200-7

**Published:** 2020-08-31

**Authors:** Zibusiso Nyati-Jokomo, Israel Mbekezeli Dabengwa, Liberty Makacha, Newton Nyapwere, Yolisa Prudence Dube, Laurine Chikoko, Marianne Vidler, Prestige Tatenda Makanga

**Affiliations:** 1 The University of Zimbabwe, College of Health Sciences, Parirenyatwa Hospital, Harare, Zimbabwe; 2 National University of Science and Technology, Faculty of Medicine, Mpilo Hospital, Bulawayo, Zimbabwe; 3 Surveying and Geomatics, Midlands State University, Senga, Gweru, Zimbabwe; 4 Research and Postgraduate Studies, Midlands State University, Gweru, Zimbabwe; 5 Department of Obstetrics and Gynaecology, The University of British Columbia, Vancouver, Canada

**Keywords:** Pregnancy, transport, RoadMApp, Barriers to maternal health services, Geographically enabled mHealth, Mobile health, Kwekwe

## Abstract

**Background:**

Travel time and healthcare financing are critical determinants of the provision of quality maternal health care in low resource settings. Despite the availability of pregnancy-related mHealth and smart travel applications, there is a lack of evidence on their usage to travel to health facilities for routine antenatal care and emergencies. There is a shortage of information about the feasibility of using a custom-made mobile technology that integrates smart travel and mHealth. This paper explores the feasibility of implementing a custom-made geographically enabled mobile technology-based tool (RoadMApp) to counter the adverse effects of long travel times for maternal care in Kwekwe District, Zimbabwe.

**Methods:**

We frame the paper using the first two steps (listen & plan) of the Spiral Technology Action Research (STAR model). The paper uses an exploratory case study design and Participatory Learning Approaches (PLA) with stakeholders (community members) and in-depth interviews with key informants (health care service providers, pregnant women, transport operators). One hundred ninety-three participants took part in the study. We conducted focus group discussions with pregnant women, women of childbearing age, men (household heads), and elderly women. The discussion questions centered on travel time, availability of transport, cellular network coverage, and perceptions of the RoadMApp application. Data were analysed thematically using Nvivo Pro 12.

**Results:**

Most parts of rural Kwekwe are far from health facilities and have an inefficient road and telecommunications network. Hence, it is hard to predict if RoadMApp will integrate into the lives of the community - especially those in rural areas. Since these issues are pillars of the design of the RoadMApp mHealth, the implementation will probably be a challenge.

**Conclusion:**

Communities are keen to embrace the RoadMApp application. However, the feasibility of implementing RoadMApp in Kwekwe District will be a challenge because of maternal health care barriers such as poor road network, poor phone network, and the high cost of transport. There is a need to investigate the social determinants of access to maternity services to inform RoadMApp implementation.

## Background

Several studies have pinpointed the optimal time taken by pregnant women to access maternal care as a predictor of maternal health outcomes [1]. There is a growing need for interventions employing Geographic Information Systems (GIS), making use of maps and geospatial analyses to improve birth preparedness and complication readiness (BPCR) [2, 3]. Health interventions to improve BPCR using GIS have the potential to integrate with online platforms (i.e., web GIS) and mobile devices or telemedicine (i.e., call and short message services) [4]. Such health interventions have the potential to reduce health costs [5] and improve health outcomes [6].

Birth Preparedness and Complication Readiness includes: (a) knowledge of pregnancy danger signs; (b) shared decision-making before the onset of labour and potential occurrence of obstetric complications; (c) planned location for delivery and emergency services; (d) transport plan; and, (e) savings plan [7]. We relate the definition of BPCR to *Thaddeus and Maine’s three delays model,* which postulates (a) delay by the individual and the family in deciding to seek care, (b) delay in reaching care, and, (c) delay in receiving care [8].

An administrative meeting with community leaders, health practitioners, and the general populace in Kwekwe District raised concern over poor maternal health statistics [9, 10] and delays by pregnant women in resource-constrained areas in reaching health centres because of transportation problems. Over two thirds (67.1%) of rural women in the District deliver at clinics/hospitals, compared to 97.4% in urban areas [11]. Rural women are more vulnerable to maternal complications (3.3% referrals in labor) [9], and there is a high national maternal mortality ratio (MMRatio) of 462 deaths per 100,000 live births [10]. Hence, the Place Alert Labs (PALs) team from the Midlands State University, Zimbabwe, conceptualized the RoadMApp application to counter the adverse effects of delayed transportation of pregnant women to health facilities. This application links pregnant women to locally available transport. PALs have a track record for developing interventions with GIS, applying maps, and geospatial analyses to improve BPCR [2, 3].

Mobile devices have the potential to reach multiple people at the same time. They have a far-reaching value in healthcare, with uses for diagnostics, health management, and telemedicine. Applying mHealth (mobile health technology) strategically solves problems related to the time, distance travelled and coordination of stakeholders [12]. However, despite the availability of pregnancy-related mHealth applications, there is a lack of evidence on how they integrate into the daily lives of pregnant women and if they influence behaviour change on BPCR [13]. This is because some mHealth applications have not been developed together with the end-user and cannot recognize the users’ context. Technology is not neutral, it is context-specific and has to be adapted to user needs to avoid low or non-usage [14].

We performed a desktop literature search to find similar mHealth interventions or studies that embody the interests of this project. Prior studies on smart travel applications focus on taxis [15] and health and geography warnings for holiday destinations [16]. Most mHealth solutions on prenatal and postnatal healthcare focus on sending health-related information on treatment adherence and disease monitoring [5, 14]. Examples of interventions that closely match the RoadMApp mHealth rely on private vehicles, geospatial location, equipment, and coordination through a call centre [17]. However, most of the features are experienced from the service providers’ side, giving no options to the end-user for the choice of driver and vehicle, and there is no telemonitoring (using information technology to monitor patients remotely).

**Fig. 1:**
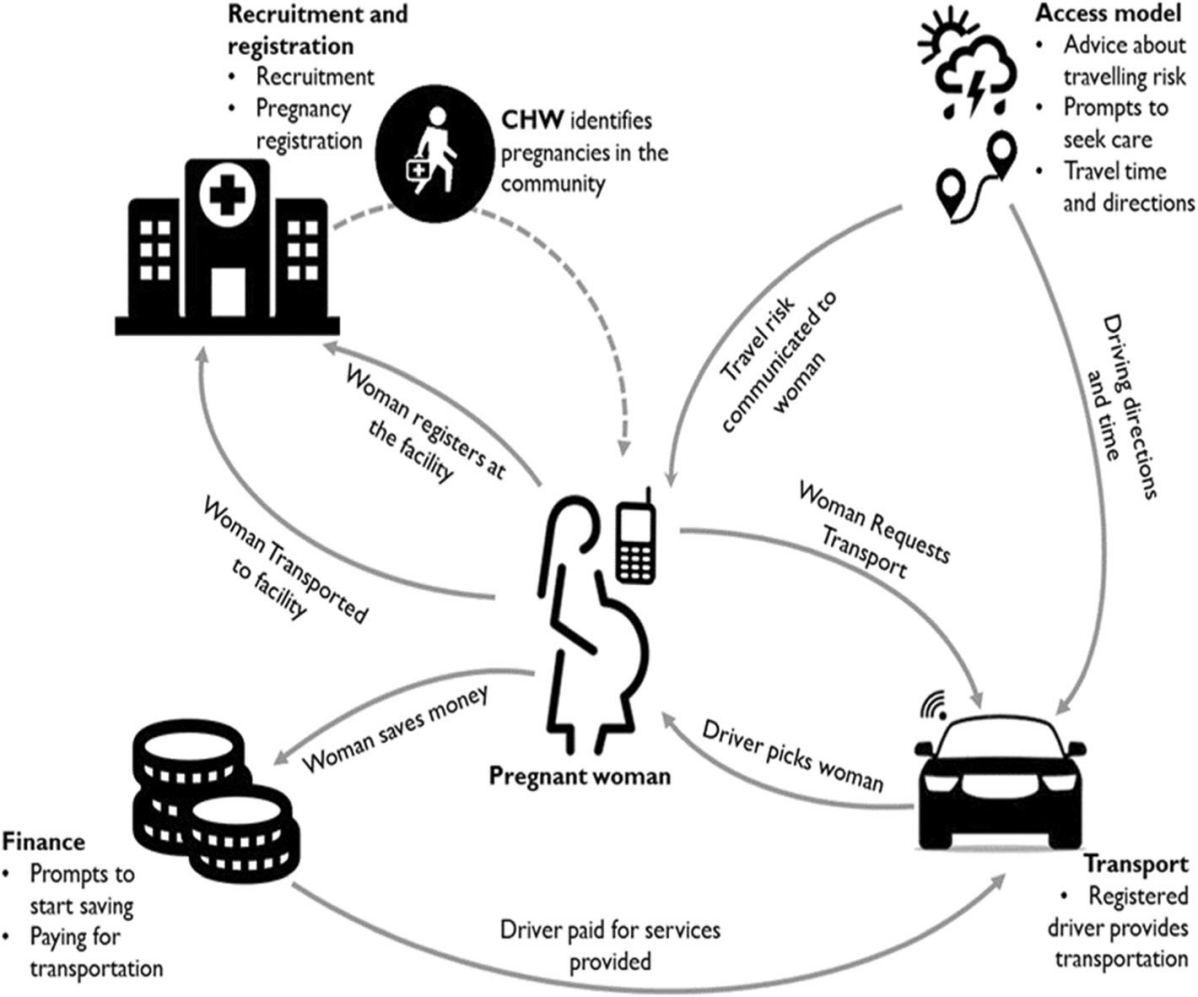
Showing an infographic of the proposed RoadMApp mHealth intervention [18]

The PALs team devised an application called the RoadMApp, which integrates smart travel and mobile health technology [18]. The intention is to ease women’s transportation challenges to health facilities by connecting them with locally available transport. An infographic of the proposed RoadMApp mHealth application is shown in Fig. 1.

The RoadMApp mHealth rides on the assumptions of a high mobile phone penetration rate in Zimbabwe [19], available transport options, long travel times for accessing maternal healthcare facilities, resulting in maternal delays and birth complications [20]. Therefore, an understanding of existing strategies and perspectives for mobilising transport and financial resources might lead to the development of an intervention that would improve maternal health outcomes.

The RoadMApp mHealth intervention will comprise four components, namely: (a) recruitment and pregnancy registration, (b) modelling geographical access to care and, (c) finance (facilitating savings to access care), and mobilising transportation to seek care.

### The objectives of the study

This paper reports part of a broader feasibility study of a mHealth tool called RoadMApp that seeks to address the most critical gaps in women’s travel to institutional birth facilities. Specifically, the paper aims to understand women’s experiences (end-users) in traveling to the institutional birth facility (context) and, to assess the coordination and resources needed to develop the RoadMApp mHealth tool.

### Theoretical framework

We frame the paper using the Spiral Technology Action Research (STAR) Model. This model integrates health promotion theory, participatory action research, critical pedagogy, software development approaches, and change strategies borrowed from the field of organizational studies [21]. It contains the following iterative and evolutionary processes concepts: listen, plan, do, act, and study. As a result, we take a qualitative deductive approach as the papers’ objectives suit the first two steps (listen & plan) of the STAR model.

## Methods

### Research design

The case study research design suits the investigation of the complexity of community concerns. We chose Participatory Learning for Action (PLA) methods to engage the community in line with the STAR model. PLA combines action and participatory action research with several techniques borrowed from qualitative social research (participatory, visual techniques such as maps, transect walks, and problem trees) [22]. The discussion questions centred on travel time, availability of transport, cellular network coverage, and participants’ perceptions of the RoadMApp application (see Additional file 1, showing the research instrument).

### Participants and setting

**Fig. 2:**
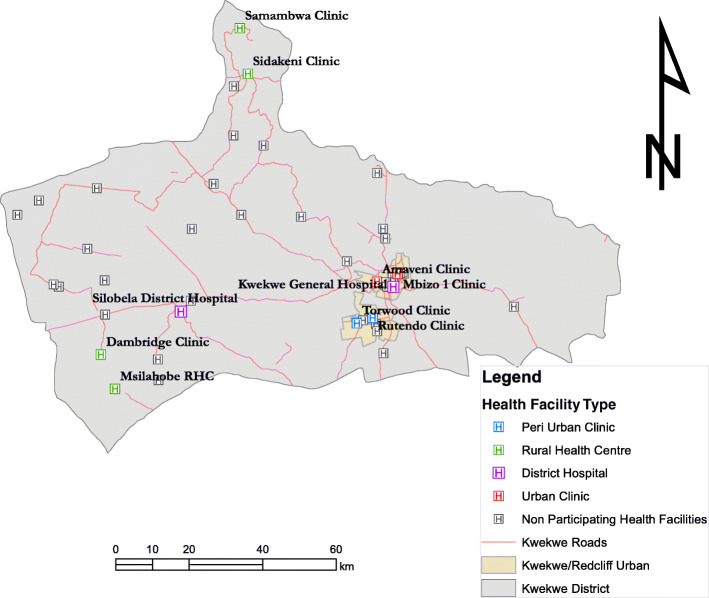
Showing the map of the data collection sites [18]

We used registers and maps at the provincial level for the selection of study sites. We purposively sampled two hospitals (rural and urban), four rural health centres, two peri-urban clinics, and two urban clinics to get information-rich cases in the communities (see Fig. 2).

We based the selection criteria on health facilities providing obstetric care services, distance to the health facilities, and availability of mothers’ waiting shelters. We treated a radius of 40 km as the catchment area of each rural clinic. We then looked for representative cases (i.e., Kwekwe residents with experience and knowledge of travelling to institutional birth facilities) at each health centre. This purposive sampling strategy accounts for participants’ clinical variation (parity), demographic groups, geographical spread (i.e., rural, peri-urban, and urban). This is consistent with the goals of qualitative research to achieve credibility and transferability as opposed to validity and generalization used in quantitative studies [23]. We asked health workers to help identify people whose experiences could significantly contribute to the study (snowballing). An interview guide was used to solicit information from the participants. The participants included pregnant women, women of childbearing age (WOCBA), community members, older women (50 years and above), and men (i.e., their roles as household decision-makers) and health care providers. Participants provided written or verbal (particularly for the less literate) informed consent.

### Data analysis

Data from the audio voice recorders were transcribed verbatim and later translated into English. Two translators verified the transcripts for authenticity. We imported the anonymized transcripts into Nvivo Pro 12™ to generate codes to aid in the analysis process. ZNJ and IMD performed first-cycle coding using elemental methods (i.e., descriptive and in vivo coding) [24]. They shared the coded data with the rest of the team to identify emerging themes. After the first-cycle, a thematic analysis summarized the data to identify and interpret the key features of the data, guided by the initial research questions [25]. Three authors cross-checked the themes with the transcripts and field notes to ensure consistency. We disseminated the preliminary findings to local authorities and the participants to verify if we had accurately represented their views.

## Results

One hundred and ninety-three (193) people took part in the study, through eleven [10] focus group discussions (FGDs), seven-teen [17] in-depth informant interviews (IDIIs), and three [3] community meetings (see Table 1 for a break-down).

**Table 1:** Study participants

Category	Rural participants	Peri-urban	Urban	Total no. of participants	Age ranges	Educational level	Marital status
**FGDS**							
Pregnant women	14	8	12	34	17–38	22 Secondary school.	26 married;
						10 Primary school.	8, not married
						2 none	
WOCBA	20	–	15	35	19–37	27 Secondary school.	23 married;
						5 Primary school.	6 widowed;
						2 none	1 single;
							5 divorced
Elderly women	17	–	7	24	53–65	11 Secondary school.	15 married;
						9 Primary school.	3 single;
						4 none	6 widowed
Spouses	–	–	8	8	23–65	8 Secondary school	8 married
**KEY INFORMANTS**							
Health staff	5	4	1	10	28–51	10 Secondary school	8 married;
							2 single
Transporters	–	1	–	1	42	Secondary school	1 married
Pregnant women	3	–	–	3	23–29	Secondary school	3 married
WOCBA	–	1	1	2	25–36	Secondary school	1 married;
							1 divorced
Spouses	–	–	1	1	43	Secondary school	1 married
**PARTICIPATORY LEARNING APPROACHES (PLA)**							
Community members	74	–	–	74	27–64	57 Secondary school.	51 married;
						12 Primary school.	19 widowed;
						5 none	4 single
**Total no. of participants**							**193**

The participants’ ages ranged from 17 to 65, with over two-thirds having attained secondary level education. Tertiary education was not prioritised in both the urban and rural settings because of the lack of opportunities that have been further compounded by the economic downturn in the country. The major sources of income were mining, both formal and artisanal. Incomes ranged between USD $20–250 per month, and there were no significant differences in the salaries between the urban and the rural dwellers. Participants in the urban area lived within a radius of 5 km to the health facilities, while two-thirds in the rural areas lived within 10 km and a third living as far as 40 km.

The PLA methods resulted in 3 key themes: (a) factors influencing the choice of transport, (b) telecommunication and network systems, (c) community perceptions of the feasibility of the RoadMApp. Table 2 presents the major themes and associated sub-themes.

**Table 2:** Themes, sub-themes, and codes resulting from the data analysis

Superordinate Theme	Sub-theme	Codes
1. Factors influencing the choice of transport	Quality of transport	• Affordability • Transport network • Safety of the transport • Waiting for time travel • Cost of travel • Ambulance system • Waiting mothers’ shelters
2. Telecommunication and network systems	Network coverage	• Mobile phone ownership
3. Community perceptions of the feasibility of the RoadMApp	Positive perceptions of the intervention	• Reducing maternal delays • Travel for referrals • Lower travel costs
	Wicked problems (economically difficult problems)	• Poor economy • Poor road infrastructure

### Theme 1: factors influencing the choice of transport

Participants defined factors influencing the choice of transport as the affordability, transport network, safety of the transport, waiting for time travel, cost of travel, ambulance system, and waiting mothers’ shelters.

### Sub-theme 1.1: affordability

Cheaper modes of transport were available in urban areas. These included the government-subsidized Zimbabwe Urban Passenger Corporation (ZUPCO) buses. Some pregnant women shared rides in Honda Fit vehicles, which carried 6–8 passengers *(instead of the standard capacity of 4) and c*ommuter buses, which took 18 passengers *(instead of 14).* The women preferred these commuters and Honda Fit taxis because of availability and shorter waiting time. The cheaper buses also had challenges of long waiting times and overloading. The following excerpt sums up the women’s experiences on the use of public transport:

*"ZUPCO is cheap but very hot (because of overloading). Passengers, drivers, and conductors seem not to understand or empathize with pregnant women. Even if I am pregnant, they expect me to stand. Sometimes when the bus is full, and they can leave you behind. However, smaller vehicles do not take time to load, but their design is not 'pregnant woman-friendly.' The poor road terrain even makes things worse,"* (An urban pregnant woman within the age range of 22-26).

### Sub-theme 1.2: the transport network

Urban areas have an established road-network as compared to Kwekwe rural. The major roads in the urban and peri-urban areas were tarred, while the minor roads are dusty with gravel and potholes. Some peri-urban places were hard to access, and most vehicle owners were reluctant to use those routes because of probable damage to their vehicles. These hard-to-access areas negatively affect the waiting time, possibly leading to home births or born before arrival, as shown in the following comment:

"*You wait for hours before they pick you as transporters would prefer to ferry people from areas where roads are good. I experienced labour pains at 9.00 am and immediately called the transporter who came 5 hours later. I could have given birth at home,"* (An urban postpartum woman within the age range of 18- 24).

### Subtheme 1.2.1: communities preference of transport options

Community meetings discussions showed a preference for locally available transporters arguing that there was a reduction of waiting time as prearrangements would have been made. Local transporters knew the road network and the accessibility of roads during different seasons. Participants said commercial transporters to be unreliable, as when the need arose, they would be elsewhere doing business. They were also reportedly charging exorbitant fees. A community member said:

"*Our people can navigate their way to the health facilities using the worst of roads, and they show a human face in that they are prepared to get the woman to the health facility irrespective of the wear and tear of their cars,"* (Female FGD participant, age range 35–40*)*

To make the RoadMApp workable, spouses suggested having a list of drivers to choose from and standardised costs.

### Sub-theme 1.2.2: safety of the transport

Despite the availability of public transport in urban areas, most pregnant women mentioned the safety and unaffordability of public transport, especially for routine visits to health facilities. This saw pregnant women within the 5-10 km range walking to the clinic/hospital. Pregnant women who walked would leave home at around 0400 h to catch the queue at the clinic/hospital *(that opened at 0700 h)*. In rural areas, the most common mode of transport is animal-driven carts (scotch carts) that are accident-prone. Sometimes, women in labour walk long distances to access the road network to get transportation. The situation is worse in the rural areas where pregnant women reportedly walk more than 20 kms.

Women both urban and rural do not feel safe to catch a ride in a car whose driver is unknown, as they had heard of criminals who were prowling most places taking advantage of unavailability or poor lighting. One older woman said:

*"It is dangerous to board a vehicle from an unknown driver as we have heard reports of people being murdered by unknown assailants,"* (FGD participant, age range 55-60).

Walking any distance during labour could be problematic in the day and worsened at night. The following excerpt summarizes the ordeal of a woman who went into labour at night:

*"I called our local transporter when my daughter got into labour, and he told me he had other errands to do, and he would only be available after 3hrs. That would have been a serious delay as the labour pain had intensified. I asked her siblings to accompany her. They walked in the dark for 2 hours before they got transport to the health facility. They could have been robbed, or she could have delivered in the forest,"* (An elderly woman within the age range 55-60).

To safeguard pregnant women, community meetings suggested a creation of a list of all eligible drivers, a community resource that would be shared with women during their antenatal classes (ANC). The older women and some men raised concerns about the cultural appropriateness of allowing men to transport women who were not their wives to the health facility. An elderly woman reiterated:

*"We have heard stories of pregnant women being abused en-route to the health facilities,"* (Participant age range 55-60).

The men gave suggestions to the team for the male spouse or relative to accompany the woman to the health facility.

### Sub-theme 1.3: cost of travel to clinics

Vehicle owners/drivers were reportedly charging high prices because they took advantage of clients who would be (a) panicking, (b) desperate, and, (c) with an acute need of transport, especially during the night. The remotest village had the highest cost of hiring a car during an emergency, with some women asked to pay an equivalent of USD$100 (Z$1000) for a distance of 20 km. The amount they paid was equivalent to fares for distances of 230 km. To justify excessive charges, a transport provider revealed that transporting pregnant women was risky as the drivers lacked skills of handling emergencies that could occur on the way to the health facilities. One transporter commented:

*"Seeing a woman delivering is not anything that a male would want to witness. By ferrying a woman in labour to the clinic, you put yourself at risk",* (Male transporter within age range 33-40).

Communities throughout the District requested for a standardised model of pricing by transporters to avoid discrepancies and overcharging. They also recommended basic emergency care training and the provision of essential medical supplies for dedicated transport operators as a way of bridging the gap where there were no ambulances.

### Sub-theme 1.4: ambulance system

Ambulances that were based at the District or General hospitals were only called in cases of emergency for both urban and rural women. The ambulances whose standard charges were the equivalent of USD$3 were overwhelmed and often delayed. The delays were further compounded by the rampant fuel shortages in the country. Ambulance drivers were said to demand only cash for their services, yet cash was not easily accessible because of the economic crisis in the country. A health worker commented on the complexity of issues about ambulance availability:

*"We only have one ambulance for all clinics. Imagine if there are complications at the same time. The ambulance is not for emergencies only. We also use it for duties like the collection of drugs from other health facilities",* (A key informant from one of the rural clinics, age range 30-35).

Local authority officials attributed the inefficiency of the ambulance system to an increase in the population, with some citing that on a single day, they could receive a maximum of 10 calls for one ambulance. The desperation for transport led to the usage of fire brigades for emergency transportation of women to the health facilities. Because of the unreliability of the ambulance services, some urban and peri-urban residents with medical aid cover used private ambulances. There were no private ambulance services in rural areas, as most of the population did not have any form of health insurance. A single ambulance in rural communities could cover a radius of over 200 km in rough terrain, and pregnant had to be transported by scotch carts or wheelbarrows to accessible places.

The unavailability of cash was worse in rural areas. Some community members highlighted that ambulance drivers demanded payment in kind (e.g., goats or chickens). The communities suggested the use of electronic mobile fund transfers for the success of the RoadMApp initiative.

### Sub-theme 1.5: waiting mothers’ shelter

The non-availability of mothers’ shelters at some rural clinics/hospitals presented a need for readily available transport systems for a referral to the next level of care, which in most cases were in urban areas that were very far from the women’s homes. The length of stay affected the women as they did not have a social support system. Some women were absconding referrals and opting for risky home deliveries. A health care worker commented:

*"Rural women are reluctant to be referred to the next level of care because of lack of accommodation and support, particularly in urban settings,*" (Female Key informant within the age range 40-45).

### Theme 2: telecommunications and network infrastructure

The telecommunications network providers have tried to ensure that most areas in Zimbabwe have network coverage. The RoadMApp intervention will heavily rely on the network accessibility and hence the importance of understanding the network patterns in the study areas.

### Sub-theme 2.1: network coverage

Participants reported network coverage to be good in urban areas where all telecommunication service providers had a substantial number of base stations. However, network challenges were experienced when there were intermittent electricity supplies, a common occurrence in Zimbabwe. The farther away rural communities were from the urban areas, the weaker the network. Despite the low network coverage in most rural settings, voice calls and messages could still be received, but there was poor Internet connectivity. The communities knew the best spots to pick up signals for communication. However, this was a challenge for nurses who had to disrupt their duties to connect to networks. One participant said:

"*At this place, we have serious network challenges, and it becomes difficult when our wives need emergency help. Sometimes one has to go up a tree to access the network,*" (Rural male participant age range 28-35).

The community suggested that the RoadMApp application should have a communication system that did not rely much on the Internet and which could use the simplest mobile phones to benefit all communities. They suggested the adoption of platforms used by local mobile money wallet operators such as Econet, Netone, and Telecel, which can be accessed by punching a set of preprogrammed numbers.

### Sub-theme 2.2: Mobile phone ownership

Discussions revealed that most people in urban settings owned mobile phones, but this was not always the case in rural areas. Failure to possess a phone in the rural areas was because of the low-income levels and unavailability of electricity. Owning a phone meant that one had to have solar charging equipment. The reduced network availability in most rural areas caused communities to rely less on mobile phones and mobile banking applications. People in rural areas were reluctant to use mobile money transactions, yet it was the only medium of exchange. Those without mobile phones requested help from friends or family members. The rural participants mentioned that community members were always willing to share their phones in cases of emergencies.

### Theme 3: community perceptions about RoadMApp

The participants perceived introducing the RoadMApp intervention as a strategic way of reducing pregnant women’s woes of delays in accessing transport and reaching health care facilities in remote rural communities. Unlike the urban areas, the catchments of some clinics or hospitals in the rural areas were as far as 40 km because of the spatial distribution of settlements, landscapes, and rivers in between. There were also cases where pregnant women in urban areas accessed health centres far from their homes because of different socio-cultural and economic realities. For example, referrals for primigravida and multiparous women and travelling to access scans or a caesarean section specialist. The cost of these regular check-ups was costly. Hence, the women were advocating for shared rides, which would cut on the waiting time and provide social support. Consideration of the community needs by the RoadMApp mHealth would increase the chances of its acceptability.

### Sub-theme 3.1 community perceptions of challenges of RoadMApp

Although communities appreciated the need for BPCR, there were some obstacles. For example, the issue of obstetric emergencies among women who were not booked for antenatal care (ANC). Women who booked late were reportedly aware of pregnancy preparation, but this awareness did not translate to preparedness because of financial constraints. Communities had limited sources of income to enable savings (due to low/unstable incomes and no sources of livelihood) and volatile costs of transport (because of macroeconomic issues). The community argued that RoadMApp would be adversely affected by the lack of savings for pregnancy. One community participant had the following to say:

"*There could be cases where a woman is alerted to report to the health facility, but she might not raise money for transportation. We are all aware that irrespective of the nonuser fees for maternal health care, pregnant women are still delaying in accessing health care,"* (Rural woman of childbearing age range 26-31).

The lack of maternal savings also affected women who booked early. The women regarded adequate savings as a panacea to generate money to pay for transport and out-of-pocket payments (OOPs). Hence, communities expected the RoadMApp intervention to handle mobile savings and bridge the gap between service providers who did not accept mobile money. In this way, the intervention would act as a health savings scheme targeting financially excluded communities. The savings were expcted to grow into investments to enable access to other services (food, medications) at the health facilities.

### Sub-theme 3.2: poor economy

Discussions revealed that the economic downturn was taking a toll on pregnant women, as most of them had become household breadwinners. Partners were often migratory labourers, leaving pregnant women to fend for their families (with the help of neighbours and relatives). Most husbands were artisanal miners from other districts or miners working underground and inaccessible during emergencies. A participant reflects this in the expert below:

*"Most of these women's husbands are illegal miners, and they go underground when they are needed the most,"* (Female participant, age range 40-45).

We can consider the economy a *“wicked problem”* (a significant societal or cultural problem that the communities, researchers, and the study cannot solve). Across all interviews, participants strongly felt that the RoadMApp intervention would be successful if there were funding attached to it. There were concerns about whether the response had enough funds to withstand the rapid hyperinflation, considering that participants would make savings in the local currency. Some even suggested that the World Bank initiative, which had introduced free maternity services, could be extended to the RoadMApp intervention.

### Sub-theme 3.3: poor road infrastructure

Another wicked problem cited by the participants was the inadequate or non-existent road infrastructure, which poses a challenge to the RoadMApp intervention. The poor road network makes most rural areas inaccessible. Women experiencing labour or complication signs cannot reach health facilities on time. Discussions during the community engagement suggested that the RoadMApp initiative should also budget for the rehabilitation of roads, particularly in the rural areas. One male participant asked:

"*We hope fixing roads is one of your plans if this noble initiative is to succeed,* " (Male spouse, age range 35-40).

## Discussion

This study investigates context-specific community requirements for the development of the RoadMApp intervention and determines the feasibility of implementing the same in a typical low resourced setting. The findings concerning distances and transport mobilisation are critical for planning mHealth interventions. However, health geography interventions to reduce MMRate and morbidity should not solely focus on transport but pay attention to (a) distal factors (terrain and meteorology); (b) proximal factors (culture, finance, autonomy, waiting time, transport options) and (c) transit factors (support, transport time, ergonomics) [26].

It is debatable if the distance travelled is the only predictor for access to healthcare facilities and subsequent usage of health interventions [27]. Women travel long distances to access care in search of better quality services [28]. This is consistent with the paper’s findings where rural and urban women travel long distances to access quality care.

The socioeconomic status of pregnant women and their caregivers influences the choice of transport that is used. This is seen through a high preference for low-cost transport run by public operators (which is often running into delays). These results are the same for both urban and rural people.

It is a common trend for antenatal educators to encourage pregnant women to have a BPCR plan that should be shared with health providers and caregivers [29]. Sometimes, when pregnant women and their caregivers cannot make adequate preparations, they use the community as a safety net in times of emergencies. Therefore, it is plausible to conclude that the socioeconomic status of pregnant women and their spouses could influence BPCR plans and the usefulness of the RoadMApp intervention. Economically disadvantaged families might incur “emergency birthing” and prefer low-cost healthcare found in government clinics and hospitals. Sometimes, the community may be unsupportive to prioritise pregnant women as they do not consider pregnancy as an illness and would not offer their seats in overloaded buses. A study in Ghana found that most expecting women were aware of potential help to reach clinics or hospitals on time but did not make any advance arrangements [30].

The findings suggest that women’s lack of advance birth arrangements would lead drivers to charge higher rates or refuse to transport pregnant women. Therefore, when implementing the RoadMApp mHealth, the team should note predictors of BPCR such as literacy levels of communities, localities, access to information, booking status of the women, and affordability [29].

Local studies have attributed late and low ANC registrations amongst Zimbabwean women to transport costs and charges for prenatal services, among other factors [31]. The overall issue which may resound is the macroeconomic environment, which makes planning during the pregnancy tougher, especially in the rural areas. Therefore, a comprehensive community mobilisation strategy is required to respond to maternal emergencies, rather than sporadic incidents of community mobilisation. Hence, the usage of this mHealth initiative may become negatively affected by the lack of birth preparations as RoadMApp relies on regular savings and transport mobilisation to be conducted on time.

There are a few ambulances in all the study sites resulting in long waiting times for pregnant women. Reports from the media suggest Kwekwe District a low number of ambulances in Zimbabwe [32]. Companies like Econet have launched mHealth-related ambulances called “Vaya.” The Vaya transport service is like commercial smart travel applications that use mobile and telecommunications to connect users with transport [15]. Also, the Insurance Council of Zimbabwe (ICZ) launched a rapid response ambulance [33]. To date, these ambulances have not expanded to cover the rural areas adequately. This could be a reluctance by the service providers to use terrible roads in rural areas. Out of the determinants for using an ambulance in a low resource setting [34], this study could only confirm distance, nature of the roads, availability of the ambulance, and the cost.

Though Zimbabwe has had one of the world’s highest mobile penetration rates since 2014, recent statistics suggest the mobile penetration rate is lessening because of the economic instability in the country [19]. This means that there has been a drop in active mobile subscribers, the Internet penetration rate, and mobile voice traffic. Perhaps this may explain the low number of people with mobile phones in rural areas.

Other challenges, such as the lack of electricity, are potential barriers to implementing RoadMApp in hard to reach communities. Because rural communities are deprived of mobile and telecommunication infrastructure, they may have developed an apathy for smart mobile technology. Smart mobile travel applications cannot function effectively without telecommunications infrastructure (including Wi-Fi hotspots) and digital literacy [15]. The unavailability of these factors in rural settings is a common challenge experienced in low resource settings [15]. Yet, there are mHealth interventions that have been implemented despite these barriers. RoadMApp may take the approach used in some studies where project teams have installed servers closer to communities to boost the signal [35].

There is a shortage of studies that support the usage of mHealth for smart travel in pregnancy in Zimbabwe. A notable local study did not test a specific mHealth intervention used in maternal care [36]. Another gap that is not fulfilled in the data is the current uses of mobile phones among the populations to assess their level of digital literacy. Without this sound evidence, it is hard to predict if RoadMApp will integrate into the lives of the community, particularly those in rural areas.

Relating to Thaddeus and Main’s three delays model, we also found that the second delay is caused by distance, the lack of standardised costs for transport used for maternal emergencies, low health savings, unavailability of transportation, medically trained transporters and quality road networks. This is a common finding in studies that apply the three-delay model [8, 37]. As a result, the mHealth would rely on private vehicles to cover the shortage of ambulances. There are exemplary models of best practice in countries like India [17]. Private-public partnerships (PPP) have paid vehicle owners to transport pregnant women for institutional birthing, while the women pay no cost [17]. The success of projects such as this relies on continuous funding, which is not guaranteed under the hyperinflationary environment.

The RoadMApp mHealth should consider the cost of long-distance travel for referrals, as women are likely to travel long distances to seek better quality care [28]. This is consistent with the paper’s findings, where rural and urban women travel further distances to access specialized care. The PALs team should have to consider this factor when implementing the intervention, as it is more effective to use high-volume transport for longer distances. Case studies that have taken this approach have negotiated fares or issued voucher tickets for intercity travel, reduced the second delay, and got positive maternal health outcomes [38, 39].

### Study limitations

A limitation of the study was that we excluded private ambulance operators from the data collection, making it difficult for the authors to transfer the findings to their context. This may affect the project in the later stages, especially when introducing the RoadMApp mHealth to groups with health insurance of scaling up to different locales.

The level of digital literacy among the respondents was not assessed [36]. Without this sound evidence, it is hard to predict if RoadMApp will integrate into the lives of the community - especially those in rural areas. The study findings cannot be generalized because of the small sample size. The collected information is, however, rich enough to advise the RoadMApp intervention.

## Conclusions

An understanding of the local context, where the RoadMApp mHealth intervention will be implemented, is fundamental. Communities are keen to embrace the RoadMApp application. However, the feasibility of implementing RoadMApp in Kwekwe District will be a challenge because of maternal health care barriers such as poor road network, poor phone network and the high cost of transport and the general hyperinflationary environment in the country. There is, therefore, a need to investigate the social determinants of access to maternity services to inform the RoadMApp implementation.

## Supplementary information

**Additional file 1.** Qualitative research design for the RoadMApp project.

## Data Availability

The corresponding author can avail the data when requested.

## Abbreviations

ANC: Antenatal Care
BPCR: Birth Preparedness and Complication Readiness
FGD: Focus Group Discussions
GIS: Geographic Information Systems
ICZ: Insurance Council of Zimbabwe
IDII: In-depth Informant Interviews
mHealth: mobile health technology
MMRatio: Maternal Mortality Ratio
OOPs: Out-Of-Pocket Payments
PALs: Place Alert Labs
PLA: Participatory Learning for Action
PPP: Private–Public Partnerships
RBF: Results–Based Financing
STAR: Model Spiral Technology Action Research
WOCBA: Women of Childbearing Age
ZUPCO: Zimbabwe Urban Passenger Corporation
