# Supplementary material for: RoadMApp: a feasibility study for a smart travel application to improve maternal health delivery in a low resource setting in Zimbabwe
Source: BMC Pregnancy Childbirth. 2020 Aug 31;20:501. doi: 10.1186/s12884-020-03200-7 (PMC7457488; doi:10.1186/s12884-020-03200-7)
Supplement: Supplementary file 1 — Additional file 1. Qualitative research design for the RoadMApp project. [file 12884_2020_3200_MOESM1_ESM.docx]

# Additional file

## Additional file 1: Qualitative research design for the RoadMApp project

Introduction

True access to healthcare is determined by the distance which people must travel to access care; the ability to pay for services, as well as having their needs met once at the facility. In most parts of Zimbabwe, especially in rural areas, distance to a health facility is a key determinant of maternal health. Understanding existing strategies and perspectives for mobilizing transport and financial resources to facilitate access to pregnancy related care (ante-natal and intrapartum) is one of the key aims of the RoadMApp project. This section outlines a plan to gain insights on the existing strategies and perspectives for mobilizing transport and financial resources to facilitate access to antenatal and intrapartum care services in Kwekwe district of Zimbabwe, through a qualitative research design. The information will be important in designing strategies to promote facility deliveries and improve maternal health. The qualitative data will be collected from information-rich population subgroups through focus group discussions and semi-structured interviews as will be outlined later.

**Objectives of Focus Group Discussions, Semi-structured Interviews and Community Engagement**

1. Focus Group Discussions (FGD) will be used to elicit community perspectives on known strategies for mobilizing transport and financial resources to facilitate access to pregnancy related care in the study area.
2. Semi-structured or In-depth Interviews (SSI/ID) will elicit local perspectives on known strategies for mobilizing transport and financial resources to facilitate access to pregnancy related care.
3. The idea of carrying out community engagement sessions is to sensitize key stakeholders in the district about the project in order to promote their full support and community participation/engagement in the project through:

- Identification and sensitization of community gatekeepers
- Identification and sensitization of key stakeholders for maternal health
- Community sensitization

1. **FOCUS GROUP DISCUSSIONS (FGDS)**

The FGDs will be conducted among information-rich population sub-groups to gain information on the existing strategies of mobilizing transport and financial resources to facilitate access to ante-natal and intrapartum care at health facilities in Kwekwe District. This information could be a range of details; opinions; and narratives of respondents on their knowledge and healthcare experiences as clients in Kwekwe district.

1. *Target and study population*

The target population for the FGDs will be all knowledgeable healthcare clients in Kwekwe district. The idea will be to recruit people who can significantly contribute to the study by sharing information on the existing strategies, experiences, as well as suggestions in mobilizing transport and financial resources to facilitate access to antenatal and intrapartum care for women in Kwekwe district. These people have been classified into sub-groups which include the following inclusion criteria:

- pregnant women
- women of child bearing age
- spouses of pregnant women and spouses for women of child bearing age
- elderly women (50 years and above)
- those who meet the above criteria, and provide written informed consent to participate in the FGD survey

1. *Sampling method, sample size and data collection*

The sampling frame will be composed of all Kwekwe residents who meet the inclusion criteria stated above. Sampling will start by the identification of sampling locations in Kwekwe districts. For the FGD survey, sampling locations will be healthcare facilities offering maternity services in Kwekwe district. Kwekwe district has 35 facilities providing maternity services (three hospitals, seven clinics in Kwekwe urban district, and twenty-five clinics in Kwekwe rural district), translating to 35 in the whole study area. One hospital, six rural health centres and three urban clinics offering maternal services will be selected for the study, using purposive sampling focusing on coming up with sampling locations of heterogeneous characteristics (easily accessible and hard to reach health centres), for both rural and urban locations. Thus, the study will be conducted in catchment areas of 10 sites providing maternal services in Kwekwe district.

Four main FGD participant subgroups are important to the study as identified before, which are (1) pregnant women; (2) women of childbearing age; (3) spouses of pregnant women as well as spouses of women of childbearing age who might not be pregnant by the study time; and (4) elderly women. The idea of coming with subgroups is to ensure homogeneity of personal characteristics within each subgroup, and heterogeneity between subgroups, for an uninhibited participation of members within focus group discussions.

FGD participants for the four subgroups will be identified from the catchment area of the facility, by conveniently identifying people who meet the eligibility criteria through the help of the community healthcare workers and environmental health technician (EHTs). Sampling participants from various sampling locations and seeking the help of healthcare workers working in the district will be done to increase chances of encountering participants in hard-to-rich areas.

FGDs with people from each subgroup will be conducted according to the principle of saturation (collecting data until no new information is emerging). Each focus group discussion will comprise of 8 participants. We anticipate conducting 10 FGDs in order to reach information saturation for each subgroup, thus translating to 40 FGDs for the four subgroups.

1. *Data collection instruments*

Data will be collected using FGD guide developed to collect information mainly on strategies in mobilizing transport and financial resources to facilitate access to antenatal and intrapartum care: a range of details; opinions and narratives of respondents on their healthcare experiences as clients in Kwekwe district.

1. SEMI-STRUCTURED INTERVIEWS WITH KEY INFORMANTS

Semi-structured interviews will be conducted to gain information on the existing strategies, challenges and recommendations in mobilizing transport and financial resources to facilitate access to ante-natal and intrapartum care at health facilities in Kwekwe District. The key informants to be requested for interview will include District administrators, Chiefs, Headmen, District Health Team (DHT), Provincial Maternal and Child Care Officer, Managers for NGOs on maternal healthcare, and Midwives from various facilities offering maternal services.

1. *Identification of Key Informants, sample size and data collection*

Key community gatekeepers who give permission to proceed with the study, will also be requested to participate in semi-structured interviews. These include the District Administrators for Kwekwe District (1), City Health Director for Kwekwe city (1), District Medical Officer (1), Chiefs (3) and Headmen (4), in Kwekwe District. In addition to these, members of the district health team for Kwekwe district (5), Midwives from various facilities in Kwekwe district (10), the Provincial Maternal and Child Health Officer (1), as well as managers of NGOs implementing maternal healthcare programs in Kwekwe district (3), will be requested to participate in semi-structured interviews. The total number of people expected to participate in semi-structured interviews is twenty-nine (29).

1. Data collection instruments

Data will be collected using interview guide developed to collect information mainly on strategies in mobilizing transport and financial resources to facilitate access to antenatal and intrapartum care: a range of details; opinions, and narratives of respondents on their experiences on antenatal and intrapartum services in Kwekwe district.

1. Protocol for administering instruments

The study will be conducted by fluent English speakers with fluency in both Shona and Ndebele and trained in quantitative and qualitative research methodologies. The FGD and Interview guides were developed in English and translated separately into Shona and Ndebele, which are the main first languages in Kwekwe district, and then back translated from Shona to English, and Ndebele to English. The FGD and Interview guides intended for use will be pretested and modified based on pre-test responses before the actual data collection. Pre-test participants will be excluded from the actual data collection.

Each FGD and semi-structured interview will be expected to last 45 to 60 minutes. All the FGD and interview responses will be audio-recorded and transcribed verbatim onto a word processing program.

The process of data collection will take 2 stages:

1. The first stage will involve locating and building rapport to gain trust and confidence with community members. During the 1^st^ stage, the FGD and interview guides intended for use will be pre-tested and modified for clarity and appropriateness according to pre-test responses before use by conducting five interviews with key informants (Headmen) and two FGDs with clients who meet the inclusion criteria.
2. The second stage will involve the actual data collection process: FGD and semi-structured interview surveys, using dates and venues preferred and pre-set by the respondents themselves.

**Data management**

Qualitative data: The data from FGDs and semi-structured interviews with key informants will be stored in form of audio-recordings (transcripts), FGD and interview notes, debriefing notes and session summaries, all complimented by some additional observational data obtained during the FGDs and interviews. After each FGD and interview session, audiotapes will be transcribed verbatim onto a word processing program. Transcripts will be marked and coded according to various areas of interest. Results will be combined onto a logbook consisting of a table to enable data to be organised according to topics of interest before analysis. All data will be stored securely and only used for the purpose of the study through the following:

1. Computer and file security which involves controlling access to folders and files through password protection and by encryption. Anonymization techniques will also be used to avoid disclosure of sensitive data, if any.
2. Physical data security which requires control of access to rooms and equipment where data (digital or physical) are held.
3. Avoiding storing sensitive data on a machine connected to an external network and avoiding using cloud file sharing services like Google docs and Dropbox, for sensitive data.

The data will be disposed of after the final report has been produced and deliberated on (2 years after the end of the study), to allow for secondary analysis that may result from new research questions emerging from the project.

**Data analysis**

Thematic analysis of themes on strategies to mobilize transport will be employed for analysing qualitative data from FGDs and interviews. This involves identifying, examining and recording themes (patterns) within the data. Themes are patterns existing within qualitative data: groups of related responses, that are useful in answering a specific research question, and which will become data analysis categories.

According to (Braun & Clarke, 2006), thematic analysis allows established and meaningful patterns to be created, through coding the qualitative data in six phases. In line with this principle, qualitative data for this study (related to barriers faced by SSA migrant participants to access Thai healthcare services and their coping mechanisms) was analysed following those 6 phases: “(1) familiarization with the data, (2) generating initial codes, (3) searching for themes among codes, (4) reviewing themes, (5) defining and naming themes, and (6) producing a final report”, Clarke (2006). Findings were presented according to issues of interest and quotations were used to illustrate expressed thoughts, emotions and experiences by participants.

1. A plan for community engagement for the RoadMApp project

Prior to the implementation of the RoadMApp project, key stakeholders in the district will be sensitized about the project in order to promote their full support and community participation/engagement in the project. The process of community engagement will be conducted in stages outlined below:

1. Identification and sensitization of community gatekeepers
2. Identification and sensitization of key stakeholders for maternal health
3. Community sensitization
4. *Identification and sensitization of community gatekeepers*

Key community gatekeepers in Kwekwe district will be identified, and sensitized on the goals of the RoadMApp project, prior to the implementation of the project. Key community gatekeepers to be sensitized include the Provincial Medical Director, District Administrator, Paramount Chiefs and Chiefs, Headmen, and City of Kwekwe Health Director.

1. *Identification and sensitization of key stakeholders*

Key stakeholder for maternal health will also be identified and sensitized on the goals of the RoadMApp projects. These include District Medical Officer and other members of the District Health Team, community health workers, and managers of NGOs implementing maternal health projects in the district.

1. *Community sensitization*

Community sensitization on the goal of the RoadMApp project and its associated activities within the community will be conducted before the start of the project and continue throughout the course of the project to its completion. Sensitization of the community will be done in consultation and collaboration with community gatekeepers and health stakeholders. Various strategies will be employed in communicating with community members about the project, including targeting members attending community gatherings like village and ward meetings, and clinic appointments like antenatal care and child vaccination visits. Village/community health workers who will have been sensitized about the project will also be tasked to cascade the information down to community members during their day-to-day duties in the community. The key communication information on the project goals, target population, project duration and expected benefits to the community will be communicated to the community and stakeholders through meetings, local radio stations and bulk short message system (SMS).

## Annexure 2: Focus Group Discussion Guide – (English)

1. Can you share your experiences on how pregnant women in this community are currently getting to the health facility for the following?
2. Antenatal care
3. Delivery

*(forms of transport, travelling time, cost to the nearest clinic, availability time, seasonal variations and effect on roads and transport costs, maternity waiting homes)*

1. What challenges are faced by people in this community in getting transport and paying for access to the health facility for the following:
2. For antenatal care services
3. To give birth
4. What are the various strategies being employed by people in this community to mobilize transport and the required financial resources for pregnant women to attend?
5. Antenatal care visits
6. delivery

*(various strategies for various transport options, payment strategies/options, financial mobilization strategies)*

1. What are the various transport options used by pregnant women to access the health facility for the following?

- antenatal care
- delivery

1. How useful are the various transport options in transporting pregnant women to the health facility for?

- antenatal care
- delivery

*(mention the various transport options, their reliability, cost, payment options and flexibility)*

1. What recommendations and suggestions would you make towards the improvement of transport services and associated financial resources for pregnant women to timeously attend for
2. Antenatal care services
3. Delivery

## Annexure 3: Focus Group Discussion Guide (Ndebele Translation)

1. liyakwanisa yini ukusitshela elahlangana lakho mayelelana ngendaba yendlela esetshenziswa ngabomama abazithweleyo abendawo leyi ukuyathola uncedo esibhedlela lalokhu okulandelayo:
   1. Ukuyakhangelwa isisu
   2. Ukuyabeletha
2. Yikuyini okuhlupha omama abazithweleyo baleyi ndawo ukuya esibhedlela kumbeni ukubhadala imali engafuneka ukuncediswa okulandelayo:
   1. Ukuyakhangelwa isisu
   2. Ukuyabeletha
3. Yikuyini okutshiyeneyo okuyenzwayo ngabantu bendawo leyi mayelelana ngendlela ezingasetshenziswa,lemali engafuneka , labomama abazithweleyo ukuya thola uncedo lalokhu okulandelayo:
   1. Ukuyakhangelwa isisu
   2. Ukuyabeletha
4. Yiziphi indlela ezitshiyeneyo ezisebenzwisa ngabomama abazithweleyo bendawo leyi ukuya esibhedlela ukuya cediswa lalokhu okulandelayo:
   1. Ukuyakhangelwa
   2. Ukuyabeletha
5. libona indlela ezisebenziswa ngabomama abazithweleyo abendawo leyi ukuya esibhedlela ziqakatheke okunganani:
   1. Ukuyakhangelwa isisu
   2. Ukuyabeletha
6. Yiwaphi amacebo elingawanika ukuthi kubeletshintshi zendlela zokukambisa Kanye lokubambanisa imali engafuneka ukuthi omama abazithweleyo bakwanise ukufika esibhedlela kusaleskhathi sokuncediswa okulandelayo:
   1. Ukuyakhangelwa isisu
   2. Ukuyabeletha

## Annexure 4: Focus Group Discussion Guide (Shona Translation)

1. Mungakwanisawo here kutaura zvamakambosangana nazvo maererano nenzira dzinoshandiswa nemadzimai akazvitakura emudunhu rino kuenda kuchipatara kunotsvaka rubatsiro runotevera
   1. Kunotarirwa pamuviri
   2. kunopona
2. Ndeapi matambudziko arikusangana nemadzimai akazvitakura emudunhu rino pakuenda kuchipatara kana pakubhadhara miripo ingadiwa kunowana rubatsiro runotevera:
   1. Kunotariswa pamuviri
   2. kunopona
3. Ndeapi mazano akasiyana siyana arikuitwa nevanhu vemudunhu rino mukuronga mafambirwo angaitwa, huye mibhadharo ingadiwa, nemadzimai akazvitakura kunowana rubatsiro runotevera
   1. pakuenda kunotariswa pamuviri
   2. pakunopona
4. Ndedzipi nzira dzakasiyana siyana dzekufambisa dzirikushandiswa nemadzimai akazvitakura emudunhu rino pakuenda kuchipatara kunowana rubatsiro runotevera
   1. kunotarirwa pamuviri
   2. kunopona
5. Munoona dzakakosha sei nzira dzakasiyana siyana dzinoshandiswa nemadzimai emudunhu rino akazvitakura mukuenda kuchipatara
   1. kunotarirwa
   2. kunopona
6. Ndedzipi kurudziro dzamungape kuti pave neshanduko yakanaka mukuvandudzira zvekufambisa pamwechete nekuunganidzwa kwemari dzingadiwa kuti madzimai akazvitakura akwanise kusvika kuchipatara paine nguva kunowana rubatsiro rwunotevera
   1. Kunotariswa pamuviri
   2. kunopona

## Annexure 5: Semi-Structured Interview Guide – (English)

1. Can you share your knowledge on how pregnant women in your area are getting to the health facility for?
2. Antenatal care
3. delivery

*(forms of transport, travelling time ranges, availability, costs, effect of seasonal variations on transport availability, cost and roads, maternity waiting homes)*

1. What are your strategies, if any, in helping to mobilize transport and financial resources for pregnant women to attend?
2. Antenatal care
3. delivery
4. What strategies by other stakeholders are in place in mobilizing transport and financial resources for pregnant women in accessing the following:
5. Antenatal care
6. Delivery
7. Can you share your experiences on the challenges you are currently facing in mobilizing transport and financial resources for pregnant women to access the following?
8. Antenatal care
9. Delivery
10. What recommendations/suggestions would you make towards improving transport and financial resource mobilization for pregnant women in accessing:
11. Antenatal care
12. Delivery

## Annexure 6: Semi-Structured Interview Guide (Ndebele Translation)

1. lingasitshela yini elikwaziyo mayelelana ngendlela ezisebenziswa ngabomama abazithweleyo ukuyathola uncedo esibhedlela lalokhu okulandelayo:
   1. Ukuyakhangelwa isisu
   2. Ukuyabeletha
2. Yiziphi indlela elizaziyo,kumbeni ezikhona , elizisebenzisa ukulungiselela izinto zokukambisa lemali engafuneka ukuthi omama abazithweleyo bekambe esibhedlela ukuyancediswa okulandelayo
   1. Ukuyakhangelwa isisu
   2. Ukuyabeletha
3. Yiziphi indlela ezisebenziswa ngabanye abantu abakhona kundaba yempilakahle yabomama ukulunguselela izinto zokukambisa Kanye ngokubambanisa imali engafuneka ukuthi omama abazithweleyo bekambe esibhedlela ukuyathola uncedo:
   1. Ukuyakhangelwa isisu
   2. Ukuyabeletha
4. Linga sitshela yini okulihluphayo nxalihlanganisa izinto zokukambisa kanye lemali engafuneka ukuthi omama abazithweleyo bekambe esibhedlela okuyathola uncedo
   1. Ukuyakhangelwa isisu
   2. Ukuyabeletha
5. Yiwaphi amacebo kumbeni imicabango elingayinika ukuthi kubeletshintshi yedlela zokukambisa Kanye lemali engafuneka ukuthi omama abazithweleyo bekambe esibhedlela ukuyathola uncedo olulandelayo:
   1. Ukuyakhangelwa isisu
   2. Ukuyabeletha

## Annexure 7: Semi-Structured Interview Guide (Shona Translation)

1. Mungagoverawo here zvamunoziva maererano nekuti madzimai akazvitakura emudunhu rino anoenda kuchipatara nenzira dzipi dzekufambisa kunowana rubatsiro rwunotevera
   1. Kunotarirwa pamuviri
   2. kunopona
2. Ndedzipi nzira, kana dziripo, dzamuri kushandisa mukuronga zvekufambisa uye mari ingadiwa kuti madzimai akazvitakura aende kuchipatara kunowana rubatsiro runotevera
   1. Kunotarirwa
   2. kunopona
3. Ndedzipi nzira dzirikushandiswa nevamwe varimunyaya dzehutano hwemadzimai mukuronga zvekufambisa uye kuwunganidza mari ingadiwa kuti madzimai akazvitakura aende kuchipatara kunowana rubatsiro
   1. Kunotarirwa
   2. kunopona
4. Mungagoverawo here matambudziko amurikusangana nawo mukuronga zvekufambisa uye mari ingadiwa kuti madzimai akazvitakura aende kuchipatara kunowana rubatsiro
   1. Kunotarirwa
   2. kunopona
5. Ndedzipi kurudziro dzamungapa, kana mazano amungapa, mukuunza shanduko yekurongwa kwezvekufambisa huye mari ingadiwa kuti madzimai akazvitakura aende kuchipatara kunowana rubatsiro runotevera
   1. Kunotarirwa pamuviri
   2. kunopona

## Annexure 8: Informed consent form – FGD, SSI and Community Engagement (English)

**INFORMED CONSENT FORM**

**PROJECT TITLE: RoadMApp: Improving access to maternal care**

**NAME OF PRINCIPAL INVESTIGATOR: Dr. Prestige Tatenda Makanga**

**PHONE NUMBER: 0785011872**

**INTRODUCTION**

My name is ……………………………………………………………………………………. I am currently working in the RoadMApp research project in Kwekwe district.

**PURPOSE OF THE STUDY**

The study seeks to understand the strategies employed by healthcare providers, stakeholders and clients in mobilizing transport and financial resources for maternal healthcare services by pregnant mothers, through understanding related experiences. Distance to a health facility is an important determinant of maternal health throughout the time of pregnancy and after. You were selected to participate in this study because you were considered useful in providing the important information needed in this study, and you could potentially benefit from the findings of this study and its recommendations. Understanding the strategies being employed in mobilizing transport and financial resources services for pregnant mothers will help in identifying existing challenges and coming up with ways of addressing these to improving maternal health in the district.

**PROCEDURES INVOLVED IN THE STUDY**

If you decide to participate in this study, you will participate in Focus Group Discussion and/or interview which may last for 45 to 60 minutes. During that interview, you will be asked questions to do with your experiences and knowledge related to transport for pregnant women to access maternal healthcare services in Kwekwe district. You are free to ask for clarification on any question anytime during and after the interview.

**DISCOMFORTS AND RISKS**

Some of the questions may be personal; you might feel uncomfortable responding to them. You are free to decline to respond to questions which you are not comfortable with. All your responses will be kept private and confidential.

**POTENTIAL BENEFITS**

There are no direct benefits you will get for participating in this study. You will get incentives for refreshments on the day of the interview. Again, you will get more information and advice on managing pregnancy related issues, after the interview.

**STUDY WITHDRAWAL**

Participating in this study is voluntary; you may withdraw from this study anytime, without risking losing healthcare services due to you from the clinic/hospital where you receive care.

**CONFIDENTIALITY OF RECORDS**

Information collected about you and your responses will be treated with confidentiality. The questionnaire to be used during the interview will be identified by a coded number instead of your name. This consent form will be separated from the coded questionnaires and stored in a secure place.

**AUTHORIZATION**

I have read this paper about the study, or it was read to me. I understand the possible risks and benefits of this study. I know being in this study is voluntary. I choose to be in this study; I know I can stop being in the study without losing any healthcare service benefits entitled to me. I will get a copy of this consent form.

__________________________________________________________________________

Participant (or Guardian) Signature Date

_________________________________________

Participant (or Guardian) Name (Printed)

___________________________________________________________________________

Researcher Signature Date

___________________________________________________________________________

Witness Signature Date

## Annexure 9: Informed consent form – FGD, SSI and Community Engagement (Ndebele Translation)

**INFORMED CONSENT FORM**

**IBIZO LENDINGISISO : RoadMApp: Improving access to maternal care**

**IBIZO LOMKHULU WOKUDINGISISA: Dr. Prestige Tatenda Makanga**

**INOMBOLO ZOCINGO: 0785011872**

**ISINGENISO**

Ibizo lami ngingu………………………………………………………………….okwakhathesi ngisebenzela iRoadmap Research project esigabeni saseKwekwe.

**UKUQAKATHEKA KOKUDINGISISA**

Ukudingisisa lokhu kujonge ukuzwisisa indlela ezisetshenziswa ngabezempilakahle labazithweleyo ukulungisisa udaba lendlela zokuhamba , izimali zokusiza omama abazithweleyo ngokuzwisisa kulabo asebadlula kikho. Umango ubaluleke kakhulu kwezempilakahle lapho umuntu ezithwele lalapho sethe wabeletha. Ukhethwe ukuba ube lilunga laleyindingisiso ngoba kubonakale ukuba ungaba lolwazi ngalokho okudingisiswayo njalo lawe ungaphathiseka uthole okuncane ngemva kwalokhu kudingisisa. Ngemva kokuzwisisa indlela zokuhambisa kunye lendlela zokuphathisa ngezimali ezisetshenziswa kubomama ukuyathola usizo kwabezempilakahle kuzakuba lula ukuba kutholakale indubo njalo kutholakale lendlela zokuphathisa kulezindubo.

**INDLELA EZILANDELWAYO LAPHO KUDINGISISWA**

Nxa ungakunakana ukuba ube lilungelo lalesisdingisiso lizahlanganelana libezinqumbi kumbe libuzwe nganye nganye lokhu kuthatha imizuzu eyi 45 to 60mins. Lapho kubuzizwana uyabe uzabuzwa okunengi okugoqela ulwazi lwakho kunye lokunye osowadlula kikho mayelana ngokuthola usizo ngendlela zokuhambisa kwabomama abazithweleyo besiya kwqabezempilakahle esigabeni saseKwekwe. Ukhululekile ukuba ubuze lapho lixoxisana kunye lalapho sokuphelile okokubuzana.

**OKUBI ESINGADIBANA LAKHO**

Eminye imibuzo ifuna ukuba ababuzwayo babuzwe ngamunye ngamunye abanye bengezwa; ngeke ukhululeke ukuba uphendule eminye imibuzo abayibuzayo. Ukhululekile ukuba ungaphenduli eminye imibuzo oyizwa ikuphatha kubi.Impendulo zakho ziyagcinwa kakuhle kungabi lomuntu obalolwazi ngayo.

**OKUHLE OSUNGAKUTHOLA NGEMVA KOKUDINGISISA**

Akulalutho olukhulu ozaluthola ngemva kokudingisisa lokhu engikwenzayo kodwa lizathi ukuphiwa amanamunede okokudla nje ngalelolanga kubuzwana imibuzo. Njalo lizathi ukwaziswa ukuthi lingphathisa njani omama abazithweleyo ngokutshiyetshiyeneyo ngemva kokubuzana imibuzo.

**UKUTSHIYANA LALOLOKHU KUDINGISISA**

Ukuba lilungelo lalokhu kudingisisa kuyazikhethelwa akukho obanjwa ngamandla ukuze abe lilunga. Uvunyelwe ukutshiya ngesikhathi ofuna ngaso lapho usenza uvikela ukuba ungakhitshwa ukuthola usizo kwabezempilakahle kumbe esibhedlela abakusiza kuso.

**IMFIHLO NGALOKHO OKUBHALISILEYO KUMBE OSAZISE KHONA**

Lokho othe wasizisa khona ngawe kuyagcinwa kakuhle akulamuntu ozakuba kwazi.Iphepha elizasetshenziswa ukukubuza lokubhala impendulo alisoza libhalwe ibizo lizafakwa amanombolo akho wedwa ukwenzela ukufihla**.** Amaphepha lawa azagcinwa kakuhle .

**UKUVUMELANA**

Ngiyavuma ukuba sengilibalile iphepha leli kumbe sengilibalelwe . Ngiyazwisisa okuhle lalokho esengingadibana lakho ngalesisidngisiso. Ngiyakwazi ukuba ukuba kulesisidingisiso kuvunyelwa njalo umuntu ezikhethela. Ngiyakhetha ukuthi ngibe Phakathi kwalokhu kudingisisa; Ngiyakwazi ngiyenelisa ukuma ukuba Phakathi kwalesisidingisiso ngingamiswanga ukuthola ukuphathiseka kwezempilakahle.

Ngizathola elami iphepha____________________________________________________________________

Ilunga kumbe omeleyo (sayina) ilanga lalamuhla:

_________________________________________

Ibizo lelunga kumbe omeleyo:

___________________________________________________________________________

Isignetsha kalowo odingisisayo ilanga lalamuhla:

___________________________________________________________________________

Isignetsha kalowo obonileyo: ilanga lalamuhla

## Annexure 10: Informed consent form – FGD, SSI and Community Engagement (Shona Translation)

**MVUMO YEKUPINDA MUTSVAKIRIDZO**

**ZITA RETSVAKURUDZO:** RoadMApp: Improving access to maternal care

**ZITA REMUKURU WETSVAKURUDZI**: Dr. Prestige Tatenda Makanga

**MBOZHANHARE**: 0785011872

**NHANGANYAYA**

Zita rangu ndinonzi **…………………………………………………………………………………………………………………**. Parizvino ndirikushanda ndiri mutsvakurudzo ye RoadMApp project irikuitwa muno mudunhu reKwekwe.

**DONZVO RETSVAKURUDZO**

Tsvakurudzo ino yakanangana nekuda kunzwisisa mabhindauko arikuitwa mudunhu rino nevashandi vezveutano, vavanoshanda vakatsigirana navo, nevagari vemudunhu rino, maererano nehurongwa hwemafambiro angaita vakadzi vanepamuviri mukuenda kuchipatara kunowana rubatsiro hwehutano hwavo nguva yavanenge vakazvitakura pamwe nekunopona. Izvi zvinogona kunzwisisika kupfurikidza nekunzwisisa zvinosangana navose ava varimunyaya dzehutano hwemadzimai akazvitakura. Mukuriro wemufambo wekubva kunogara vanhu kuenda kuchipatara wakakosha zvikuru pakududzira hutano wevakadzi vanepamuviri kubva munguva yekuzvitakura kusvika panguva yekupona. Masarudzwa kupinda mutsvakurudzo ino nokuti maonekwa makakosha mukupa ruzivo rwakakosha hunodihwa mutsvakurudzo ino, huye munogona kungobatsirikana nezvichazowanikwa nekukomekedzwa kubva mutsvakurudzo ino. Kunzwisisa mabhindauko anoitwa mudunhu reKwekwe maererano nehurongwa hwemafambiro anoitwa nemadzimai anepamuviri kwakakoshera kuwona matambudziko aripo nekuwana nzira dzingashandiswa pakusimudzira hutano hwemadzimai akazvitakura mudunhu rino.

**ZVINOITWA MUTSVAKURUDZO INO**

Kana abvuma kupinda mutsvakurudzo ino, muchapinda nhaurirano yegungano revanhu gumi hana kubvunzwa mibvunzo moga zvingangotora maminitsi makumi mana kusvika pamakumi matanhatu. Munhaurirano iyi, muchabvunzwa maererano maererano nezvamunosangana nazvo neruzivo hwenyu maererano nemufambiro unoita madzimai anepamuviri kunowana rubatsiro rweutano hwavo kuzvipatara mudunhu reKwekwe. Makasununguka kukumbira kujekeserwa pose pamunenge musinga kunzwisisa.

**KUSAGADZIKANA KANA NJODZI**

Mimwe mibvunzo ingangova yakavanzika; mungasangosununguka kuipindura. Sunungukai kuramba kupindura mibvunzo yamusina kusununguka kupindura. Davidzo dzenyu dzose dzichachengetedzwa zvakavanzika.

**MURIPO WEKUPINDA MUSARUDZO**

Hapana muripo wakanangana nekupinda musarudzo ino wamungawane. Mungangowana kudya kwemasikati zuva retsvakurudzo. Pamusoro pazvo, munozowana ruzivo nemazano maererano nemabatiro amungaita hutano hwenyu nguva yekuzvitakura, mukupera kwenhaurirano.

**KUBUDA MUSARUDZO**

Kupinda musarudzo ino madiro enyu moga; mungangobuda pamadira pasina kurasikirwa nemukana wekubtsirwa pakirinika sezvamaisiitwa.

**KUCHENGETEDZEKA KWEMAGWARO**

Zvamuchadavidza zvichachengetedzwa zvakavanzika. Mibvinzo yamuchabvunzwa ichabviswa mazita kwoshandiswa manhamba pachinhambo chemazita. Bepa rino richaparadzaniswa neremibvunzo, zvochengetedzwa zvakasimba.

**MVUMO**

Ndaverenga bepa rino, kana kuti ndaverengerwa. Ndinozwisisa njodzi kana zvakanakira tsvakurudzo ino. Ndinoziva kuti kupinda mutsvakurudzo ino madiro angu. Ndinoziva kuti ndingangobuda mutsvakurudzo ino pasina kumiswa kwerubetsero rwandaisiwana pa kirinika ino. Ndichapiwawo bepa rangu serino

_____________________ Date___________________

Runyoro rwemupinduri/Kana mumiririri

____________________

Zita rakazara remupinduri/ Kana mumiririri

___________________ Date__________________

Runyoro rweMutsvakiridzi

_______________________ Date__________________

Runyoro rwemuchuchisi

## Annexure 11: Informed consent form – RoadMApp Pregnancy Registration – (English)

**INFORMED CONSENT FORM**

**PROJECT TITLE:** RoadMApp – Improving access to maternal health care

**NAME OF PRINCIPAL INVESTIGATOR:** Dr PRESTIGE TATENDA MAKANGA

**PHONE NUMBER: +263 785 011 872**

**INTRODUCTION**

My name is …………………………………………………………………………………………………………………… I am currently working in the RoadMApp research project in Kwekwe district.

**PURPOSE OF THE STUDY**

The study seeks to link pregnant women, their spouses and newly born kids attending antenatal care in Kwekwe District to locally available transport options. The women and their spouses will also be encouraged to save funds for purposes of accessing care through a communal micro savings scheme. Distance to a health facility is an important determinant of maternal health throughout the time of pregnancy and after. You were selected to participate in this study because you were considered to potentially benefit from the intervention provided by this study, you could potentially benefit from participating in this study or its recommendations, since you will be informed of transport availability, conditions of roads as well as by saving towards maternal health care seeking. Understanding the challenges and strategies being employed in mobilizing transport and financial resources services for pregnant mothers will help in identifying existing challenges and coming up with ways of addressing these to improving maternal health in the district.

**PROCEDURES INVOLVED IN THE STUDY**

If you decide to participate in this study, you will be asked to register your pregnancy at a health facility of your choice. During the facility registration some members from the RoadMApp project working closely with health workers at the clinic will ask some questions to understand care seeking behaviours and saving practices in your area. These interviews may last for 45 to 60 minutes, including the regular pregnancy registration procedures that are required by the health facility. During that interview, you will be asked questions to do with your experiences and knowledge related to transport for pregnant women to access maternal healthcare services in Kwekwe district. You are free to ask for clarification on any question anytime during and after the interview. In the event of any adverse event you can contact our specialist obstetrician on contact numbers 077…………………………. or 071………………………… or 073………………………………… either directly or through our call back facility.

**DISCOMFORTS AND RISKS**

Some of the questions may be personal; you might feel uncomfortable responding to them. You are free to decline to respond to questions which you are not comfortable with. All your responses will be kept private and confidential.

**POTENTIAL BENEFITS**

There are no direct benefits you will get for participating in this study. You will get incentives for refreshments on the day of the interview. Again, you will get more information and advice on managing pregnancy related issues, after the interview.

**STUDY WITHDRAWAL**

Participating in this study is voluntary; you may withdraw from this study anytime, without risking losing healthcare services due to you from the clinic/hospital where you receive care.

**CONFIDENTIALITY OF RECORDS**

Information collected about you and your responses will be treated with confidentiality. The questionnaire to be used during the interview will be identified by a coded number instead of your name. This consent form will be separated from the coded questionnaires and stored in a secure place.

**AUTHORIZATION**

I have read this paper about the study, or it was read to me. I understand the possible risks and benefits of this study. I know being in this study is voluntary. I choose to be in this study; I know I can stop being in the study without losing any healthcare service benefits entitled to me. I will get a copy of this consent form. In the event of any adverse event please contact Dr. Mushangwe on 0717129699.

__________________________________________________________________________________

Participant (or Guardian) Signature Date

_________________________________________

Participant (or Guardian) Name (Printed)

_________________________________________________________________________

Researcher Signature Date

_________________________________________________________________________

Witness Signature Date

## Annexure 12: Informed consent form – RoadMApp Pregnancy Registration – (Ndebele Translation)

**INFORMED CONSENT FORM**

**IBIZO LOKUDINGISISA** : RoadMApp – Ukuphathisa omama abazithweleyo ukuba bathole usizo ngezempilakahle

**IBIZO LIKAMKHULU WALABO ABADINGISISAYO: Dr PRESTIGE TATENDA MAKANGA**

**INOMBOLO ZOCINGO: +263 785 011 872**

**ISINGENISO**

Ibizo lami ngingu …………………………………………………………………………………………………. okwakhathesi ngisebenzela iRoadmap Research project esigabeni saseKwekwe.

**UKUQAKATHEKA KOKUDINGISISA**

Ukudingisisa lokhu kujonge ukuhlanganisa omama abalwabantwana lamadoda abo kunye Labantwana babo abathola usizo kwabazempilakahle mayelana ngendlela zokuhambisa ezitholakala e Kwekwe. Omama kunye labomkabo bazakhuthazwa ukuba begcine kanjani izimali zabo eziphthelane lokuyathola usizo kwabempilakahle lapho bephiwa ukuba ezigabeni zabo bebambane bephathisane . Umango ubaluleke kakhulu kwezempilakahle lapho umuntu ezithwele lalapho sethe wabeletha. Ukhethwe ukuba ube lilunga laleyindingisiso ngoba kubonakale ukuba ungaba lolwazi ngalokho okudingisiswayo njalo lawe ungaphathiseka uthole okuncane ngemva kwalokhu kudingisisa njengoba lizaziswa ngokubakhona kwendlela zokuhambisa kunye lesimo sendlela okuhanjwa ngazo. Ngemva kokuzwisisa indlela zokuhambisa kunye lendlela zokubphatisa ngezimali ezisetshenziswa kubomama ukuyathola usizo kwabezempilakahle kuzakuba lula ukuba kutholakale indubo njalo kutholakale lendlela zokuphathisa kulezindubo esigabeni saseKwekwe.

**INDLELA EZILANDELWAYO LAPHO KUDINGISISWA**

Nxa ungakunakana ukuba ube lilunga lalokhu kudingisiswa uzacelwa ukuba ubhalise ukuzithwala kwakho akukhathalekile kuyiphi indawo eyezempilakahle. Lapho kubhaliswa ukuzithwala lokhu , izimeli ze RoadMApp project bazakube bebuza lalabo abasebenzela ezibhedlela zakhonapho kubuzwa imibuzo befuna ukuzwisisa ngokuziphatha kwalabo abathola usizo endaweni yonaleyo.Lokhu kuthatha imizuzu eyi 45 to 60mins kugoqela ukubhalisa ukuzithwala okujayekileyo ezibhedlela kunye lakubezempilakahle. . Lapho kubuzizwana uyabe uzabuzwa okunengi okugoqela ulwazi lwakho kunye lokunye osowadlula kikho mayelana ngokuthola usizo ngendlela zokuhambisa kwabomama abazithweleyo besiya kwqabezempilakahle esigabeni saseKwekwe. Ukhululekile ukuba ubuze lapho lixoxisana kunye lalapho sokuphelile okokubuzana.

**OKUBI ESINGADIBANA LAKHO**

Eminye imibuzo ifuna ukuba ababuzwayo babuzwe ngamunye ngamunye abanye bengezwa; ngeke ukhululeke ukuba uphendule eminye imibuzo abayibuzayo. Ukhululekile ukuba ungaphenduli eminye imibuzo oyizwa ikuphatha kubi.Impendulo zakho ziyagcinwa kakuhle kungabi lomuntu obalolwazi ngayo.

**OKUHLE OSUNGAKUTHOLA NGEMVA KOKUDINGISISA**

Akulalutho olukhulu ozaluthola ngemva kokudingisisa lokhu engikwenzayo kodwa lizathi ukuphiwa amanamunede okokudla nje ngalelolanga kubuzwana imibuzo. Njalo lizathi ukwaziswa ukuthi lingphathisa njani omama abazithweleyo ngokutshiyetshiyeneyo ngemva kokubuzana imibuzo.

**UKUTSHIYANA LALOLOKHU KUDINGISISA**

Ukuba lilungelo lalokhu kudingisisa kuyazikhethelwa akukho obanjwa ngamandla ukuze abe lilunga. Uvunyelwe ukutshiya ngesikhathi ofuna ngaso lapho usenza uvikela ukuba ungakhitshwa ukuthola usizo kwabezempilakahle kumbe esibhedlela abakusiza kuso.

**IMFIHLO NGALOKHO OKUBHALISILEYO KUMBE OSAZISE KHONA**

Lokho othe wasizisa khona ngawe kuyagcinwa kakuhle akulamuntu ozakuba kwazi.Iphepha elizasetshenziswa ukukubuza lokubhala impendulo alisoza libhalwe ibizo lizafakwa amanombolo akho wedwa ukwenzela ukufihla**.** Amaphepha lawa azagcinwa kakuhle .

**UKUVUMELANA**

Ngiyavuma ukuba sengilibalile iphepha leli kumbe sengilibalelwe . Ngiyazwisisa okuhle lalokho esengingadibana lakho ngalesisidngisiso. Ngiyakwazi ukuba ukuba kulesisidingisiso kuvunyelwa njalo umuntu ezikhethela. Ngiyakhetha ukuthi ngibe Phakathi kwalokhu kudingisisa; Ngiyakwazi ngiyenelisa ukuma ukuba Phakathi kwalesisidingisiso ngingamiswanga ukuthola ukuphathiseka kwezempilakahle. Uma kwenzeka noma yisiphi isenzakalo esibi sicela uxhumane noDkt. Mushangwe ngo-0717129699

Ngizathola elami iphepha

____________________________________________________________________

Ilunga kumbe omeleyo (sayina) ilanga lalamuhla:

_________________________________________

Ibizo lelunga kumbe omeleyo:

___________________________________________________________________________

Isignetsha kalowo odingisisayo ilanga lalamuhla:

___________________________________________________________________________

Isignetsha kalowo obonileyo: ilanga lalamuhla

## Annexure 13: Informed consent form – RoadMApp Pregnancy Registration – (Shona Translation)

**MVUMO YEKUPINDA MUTSVAKIRIDZO**

**ZITA RETSVAKURUDZO: RoadMApp: Improving access to maternal care**

**ZITA REMUKURU WETSVAKURUDZO**: Dr. Prestige Tatenda Makanga

**MBOZHANHARE**: **+263 785 011 872**

**NHANGANYAYA**

Zita rangu ndinonzi…………………………………………………………………………………………………... Parizvino ndirikushanda ndiri mutsvakurudzo ye RoadMApp irikuitwa muno mudunhu reKwekwe.

**DONZVO RETSVAKURUDZO**

Tsvakurudzo ino yakanangana nekuda kunzwisisa mabhindauko arikuitwa mudunhu rino nevashandi vezveutano, vavanoshanda vakatsigirana navo, nevagari vemudunhu rino, maererano nehurongwa hwemafambiro angaita vakadzi vanepamuviri mukuenda kuchipatara kunowana rubatsiro rwehutano hwavo nguva yavanenge vakazvitakura pamwe nekunopona. Izvi zvinogona kunzwisisika kupfurikidza nekunzwisisa zvinosangana navose ava varimunyaya dzehutano hwemadzimai akazvitakura. Mukuriro wemufambo wekubva kunogara vanhu kuenda kuchipatara wakakosha zvikuru pakududzira hutano wevakadzi vanepamuviri kubva munguva yekuzvitakura kusvika panguva yekupona. Masarudzwa kupinda mutsvakurudzo ino nokuti maonekwa makakosha mukupa ruzivo rwakakosha rwunodiwa mutsvakurudzo ino, uye munogona kungobatsirikana nezvichazowanikwa nekukomekedzwa kubva mutsvakurudzo ino. Kunzwisisa mabhindauko anoitwa mudunhu reKwekwe maererano nehurongwa hwemafambiro anoitwa nemadzimai anepamuviri kwakakoshera kuwona matambudziko aripo nekuwana nzira dzingashandiswa pakusimudzira hutano hwemadzimai akazvitakura mudunhu rino.

**ZVINOITWA MUTSVAKURUDZO INO**

Kana muchinge mabvuma kupinda mutsvakurudzo ino, muchakurudzirwa kuti munyorese kuti makazvitakura pachipatara chamakasununguka kunyoresa. Pamuchanyoresa, vakamirira chirongwa cheRoadMApp vachishanda pamwe chete nevehutano vepachipatara chamunenge masarudza vachange vachibvunza mibvunzo yekuda kunzwisisa zvinoitwa nemadzimai emudunhu rino akazvitakura pakufamba kunotsvaga rubatsiro kuchipatara uye ngoro dzemoto dzamunoshandisa nemawanikirwo adzo. Vachabvunzawo mibvunzo iri maringe nekuunganidza mari yekuzobhadhara miripo ingadiwe pakufamba uye pakupihwa rubatsiro muzvipatara. Chirongwa chekubvunzwa pachipatara chinogona kutora nguva ingaite maminitsi makumi mana nemashanu kusvika pamaminitsi makumi matanhatu. Nguva iyoyi inosanganisira nguva yavana mukoti yavanenge vachida kunzwisisa zviri maringe nepamuviri zvavanobvunzurudza nguva dzose dzamunouya kuchipatara. Munhaurirano iyi, muchabvunzwa maererano nezvamunosangana nazvo neruzivo hwenyu maererano nemufambiro unoita madzimai anepamuviri kunowana rubatsiro rweutano hwavo kuzvipatara mudunhu reKwekwe. Makasununguka kukumbira kujekeserwa pose pamunenge musina kunzwisisa. Kana pachinge paitika njodzi munogona kubata chiremba akamirira chirongwa cheRoadMApp panhamba dzenhare dzinoti 0717129699.

**KUSAGADZIKANA KANA NJODZI**

Mimwe mibvunzo ingangova yakavanzika; mungasangosununguka kuipindura. Sunungukai kuramba kupindura mibvunzo yamusina kusununguka kupindura. Davidzo dzenyu dzose dzichachengetedzwa zvakavanzika.

**MURIPO WEKUPINDA MUSARUDZO**

Hapana muripo wakanangana nekupinda musarudzo ino wamungawane. Mungangowana kudya kwemasikati zuva retsvakurudzo. Pamusoro pazvo, munozowana ruzivo nemazano maererano nemabatiro amungaita hutano hwenyu nguva yekuzvitakura, mukupera kwenhaurirano.

**KUBUDA MUSARUDZO**

Kupinda musarudzo ino madiro enyu moga; mungangobuda pamadiro pasina kurasikirwa nemukana wekubatsirwa pakirinika sezvamaisiitwa.

**KUCHENGETEDZEKA KWEMAGWARO**

Zvamuchadavidza zvichachengetedzwa zvakavanzika. Mibvinzo yamuchabvunzwa ichabviswa mazita kwoshandiswa manhamba pachinhambo chemazita. Bepa rino richaparadzaniswa neremibvunzo, zvochengetedzwa zvakasimba.

**MVUMO**

Ndaverenga bepa rino, kana kuti ndaverengerwa. Ndinozwisisa njodzi kana zvakanakira tsvakurudzo ino. Ndinoziva kuti kupinda mutsvakurudzo ino madiro angu. Ndinoziva kuti ndingangobuda mutsvakurudzo ino pasina kumiswa kwerubetsero rwandaisiwana pa kirinika ino. Ndichapiwawo bepa rangu serino

_____________________ Date___________________

Runyoro rwemupinduri/Kana mumiririri

____________________

Zita rakazara remupinduri/ Kana mumiririri

___________________ Date__________________

Runyoro rweMutsvakiridzi

_______________________ Date__________________

Runyoro rwemuchuchisi

## Annexure 14: **Information Collected during Pregnancy Registration at the Facility** (English Version)

1. The RoadMApp ID
2. Address
3. ID of the health worker
4. Date of registration
5. Name of respondent
6. How is the pregnant woman related to the respondent?
7. Name of the woman
8. Date of birth of the woman
9. Level of education of the woman?
10. Marital status of the woman and type of union?
11. How is the woman related to the head of household?
12. Religion of the woman?
13. Occupation of the pregnant woman?
14. Level of education of her husband/partner?
15. Occupation of the husband/partner?
16. Is the Expected date of delivery known?
17. If the expected date of delivery is known, what is the expected date of delivery?
18. Was date of delivery determined by ultrasound scan by dates or otherwise?
    - 1. Ultrasound scan (b) Otherwise
19. Approximately how many completed months has the woman been pregnant?
20. Date of last menstrual period
21. Where is it planned for the baby to be delivered?
22. Does she plan to have a skilled birth attendant?
23. Has/did the decision-maker give(n) her permission to go for regular antenatal care visits during this pregnancy?
24. If she has an urgent/emergency problem during pregnancy and the decision-maker is not available, who will make the decision for her to seek care?
25. Is the primary decision-maker required to accompany her when she seeks care?
26. Does she have a transportation plan (arranged transport) for seeking care either for pregnancy complications or delivery and if this plan is in place, how would she get there?
27. Does the woman plan to use a maternity waiting home at the facility where she intends to deliver her baby?

## Annexure 15: **Information Collected during Pregnancy Registration at the Facility** (Shona Version)

1. Nhamba dzemukadzi muchirongwa cheRoadMApp
2. Kero yekumba kwemukadzi akazvitakura
3. Nhamba dzemunhu anoshanda mune zveutano
4. Zuva rekunyoresa pamuviri pachipatara
5. Zita remunhu ari kupindura mibvunzo
6. Munhu ari kupindura mibvunzo ane hukama hupi nemunhu ane pamuviri?
7. Zita remukadzi akazvitakura
8. Zuva rekuzvarwa remunhu akazvitakura
9. Danho refundo remudzimai akazvitakura?
10. Wanano yemukadzi akazvitakura uye rudzi rwewanano?
11. Hukama hwemukadzi akazvitakura nemuriritiri wemba yaanogara?
12. Chitendero chemukadzi akazvitakura?
13. Basa rinoitwa nemukadzi akazvitakura kuzviraramisa kana kuraramisa mhuri?
14. Danho refundo remurume wemukadzi akazvitakura kana waanofambidzana naye?
15. Basa rinoitwa nemurume wemukadzi akazvitakura kuzviraramisa kana kuraramisa mhuri?
16. Zuva rekupona kwemukadzi akazvitakura rave kuzivikanwa here?
17. Zuva ramunofungidzira kuti mukadzi akazvitakura achapona?
18. Zuva rekupona iri makariziviswa seiko?
19. Mukufungidzira kwenyu mudzimai akazvitakura ave nenguva yakadii aine pamuviri?
20. Zuva rekutanga kutevera kwemukadzi akazvitakura paakapedzisira kutevera?
21. Ko mukadzi akazvitakura achaenda kunoponera kupi kana nguva yake yasvika?
22. Ko mukadzi akazvitakura achatsvaga rubatsiro rwemunhu akadzidza zvehutano here kana nguva yake yekupona yasvika?
23. Munhu anopa mvumo kuenda kunoonekwa navana chiremba kuchipatara akabvuma here kuti mudzimai akazvitakura atsvage rubatsiro urwu?
24. Kana mudzimai akazvitakura achinge awirwa nedambudziko rinoda kuti aende kunoona chiremba munhu anopa mvumo asipo, munhu uyu akazvitendedza here?
25. Munhu anopa mvumo yekuenda kunoona vana chiremba anofanira kuendawo here nemudzimai akazvitakura kana aching oenda kuchipatara kunobatsirwa?
26. Mukadzi akazvitakura ane hurongwa hwaakaita here kuti awane chekufambisa kuenda kuchipatara kunotariswa kana kunopona uye kana hurongwa uhu huripo hwakamira seiko?
27. Mukadzi akazvitakura anotarisira kuenda kunogarira kudzimba dzinogara vakadzi vane pamuviri vanenge vave padyo nekuda kusununguka here

## Annexure 16: **Information Collected during Pregnancy Registration at the Facility** (Ndebele Version)

1. Inombolo yowesifazane okhulelwe kuhlelo LweRoadmap
2. Ikheli yowesifazana okhulelwe
3. Inombolo yesisebenzi sezempilo
4. Usuku lokubhalisa ukuzithwala esibhedlela
5. Ibizo lomphenduli
6. Sinjani isihlobo somfazi ozithweleyo lomphenduli
7. Ibizo lomfazi
8. Usukulokuzalwa lomfazi
9. Izinga lemfundo yowesifazane
10. Isimo somshado lomhlobo lokuzihlanganisa
11. Umfazi ozithweleyo uhlobane kanjani lenhloko yendlu
12. Inkolo yowesifazana
13. Umsebenzi wesifazane okhulelwe
14. Izinga lemfundo womyeni/umhlanganyeli
15. Umsebenzi womyeni /umhlanganyeli
16. Luyaziwa yini usuku olulinganisiwe ukubeletha?
17. Usuku olulingasiwe ukubeletha
18. Lokhu kwakunqunywa kanjani?
19. Kungaba zinyanga ezingaki ezigcweleyo umama ezithwele
20. Usuku lokugcina lokuya esikhathini
21. Lilungiselela ukuthi lizobelethelangaphi?
22. Uhlela ukuba lomsizi wokubeletha onekhono yini?
23. Umenzi wesinqumo usemnikezile na owesifazana imvumo yokuthola unakekelo lokubeletha ngesikhathi lesi sokukhulelwa?
24. Nxa owesifazane elenkinga yokukhulelwa ephuthumayo umenzi wesinqumo engekho ,ngubani ozokwenza isinqumo sokuba ahambe ayothola usizo lokhulelwa
25. Kuyadingakala na ukuba umenzi wesinqumo amkhaphe owesifazane nxa esiyothola usizo lokhulelwa
26. Owesifazane okhulelwe sewenzile na amalungiselelo wokuthola indlela zokuhambisa ukuyothola usizo lokukhululeka kumbe usizo lwezinkinga zokukhulelwa

27. Ingabe lo wesifazane uhlela ukusebenzisa ikhaya lokulinda lomama endaweni lapho ehlose ukuletha khona umntwana wakhe?
